# Developmental sequence of young children's understanding of “knowing,” “forgetting,” and “remembering”

**DOI:** 10.3389/fpsyg.2025.1626407

**Published:** 2025-11-20

**Authors:** Izumi Uehara

**Affiliations:** 1 Institute for Education and Human Development, Ochanomizu University, Bunkyo, Tokyo, Japan; 2 Department of Psychology, Ochanomizu University, Bunkyo, Tokyo, Japan

**Keywords:** young children, knowing, forgetting, remembering, memory-related words, metamemory, development

## Abstract

This study focused on examining young native Japanese children's linguistic understanding of words associated with awareness of one's memory, such as “know,” “forget,” and “remember,” to explore the early developmental process of metamemory. To assess whether 4-, 5- and 6-year-olds understand these words, we created new tasks for “know” and “remember,” and used a modified episodic task for “forget,” adapted from a question used in previous research concerning the state of “placing something and forgetting it.” A total of 114 children, with 38 children in each age group, participated in this study. More than 80% of the 6-year-olds understood the mental states of “knowing,” “forgetting,” and “remembering,” whereas 4-year-olds did not. The performance of 5-year-olds fell between that of the 4- and 6-year-olds, with half failing to understand “remembering” and 30% failing to understand “forgetting.” These findings indicate a developmental progression in understanding, in the order of “know,” “forget,” and “remember.” The tasks developed in this study will contribute to future research on cognitive development, as well as to educational and support practices for young children.

## 1 Introduction

Metamemory refers to the ability to monitor, evaluate, and regulate memory-related cognitive activities and behaviors based on awareness and understanding of one's own memory states. Metamemory typically emerges around ages 4–5, and continues to develop throughout the elementary school years (Godfrey et al., 2023; Schneider, 2015), although the developmental processes during this period remain poorly understood. Metamemory is closely associated with learning and daily memory-related behaviors. Understanding the early development of metamemory is essential for supporting these functions from a young age. Metamemory develops from an awareness of one's memory, and words associated with this awareness include “know,” “forget,” and “remember.” This study focused on examining children's understanding of these words to explore the early development of metamemory.

Children typically begin to use cognitive words (e.g., “know,” “remember,” “forget,” “think,” and “guess”) around ages 3–4 (Bartsch and Wellman, 1995; Bretherton and Beeghly, 1982; Shatz et al., 1983). The extent to which children understand the meanings of these cognitive words has been examined using conversational analyses or structured tasks. However, phrases such as “I remember that…,” “I think that…,” and “You know” are often used at the beginning of statements or as discourse markers, which makes it difficult to determine whether children truly comprehend the words themselves (Cherney, 2003; Miscione et al., 1978). Although children can use syntactic and structural cues to infer verb meanings (Fisher et al., 2020; Gillette et al., 1999; Papafragou et al., 2007), expressions such as “I remember that…,” “I think that…,” and “You know” can function even when the literal meanings of “remember,” “think,” and “know” are ignored, making it particularly challenging to determine when children acquire a genuine understanding of these mental state terms.

Children's comprehension of cognitive terms has often been assessed using the “hidden object” task, in which researchers examine whether children appropriately select words that describe their cognitive state in situations where they were either shown (informed) or not shown (uninformed) an object being hidden before or during the test (Johnson and Wellman, 1980; Miscione et al., 1978). For example, Moore et al. (1989) asked children to decide which of two boxes they thought contained candy based on a puppet's statements that included the terms “know,” “think,” or “guess.” Their results indicated that the distinction between “know” and “think,” and between “know” and “guess,” was not yet fully established at age 5, and that differentiating between “think” and “guess” remained difficult even at age 8. Similarly, Lockl and Schneider (2006) presented children with 12 illustrated episodes and asked them to choose from two options—such as “know,” “guess,” “remember,” “forget,” or “understand”—to identify the mental state of the character in each episode. Their results showed that the proportion of correct responses for roughly two-thirds of the cognitive words examined did not reach 50% at age 5 and did not reach 70% at age 6. Despite these efforts, the age at which children reliably understand these memory-related terms remains unclear.

Even less is known about the development trajectory of the cognitive terms “know,” “forget,” and “remember” in Japanese. Research in this area has typically relied on counting the frequency of these words in children's conversational utterances (Iwata, 1999; Sonoda and Muto, 1996). Such studies have also shown that Japanese adults use these terms less frequently than English speakers (Toyama, 2007; Naito and Koyama, 2006). Consequently, the age at which each term first emerges has remained unclear. Uehara (2014) conducted a longitudinal study of eight children, analyzing both utterance data from interviews and checklist data completed by mothers. The results indicated that use of “know” appeared around ages 2–3, whereas “remember” and “forget” began to appear around ages 4–4.5. However, the age at which children actually come to understand the meanings of these words remains ambiguous. Based on both overseas and Japanese studies, even if children begin to use cognitive terms at around ages 3 or 4, it is unclear whether true comprehension has been achieved by ages 5 or 6, suggesting that the age of acquisition may vary across different terms. Moreover, because previous tasks such as the hidden object task and word-choice paradigms assume a certain level of situational understanding and verbal reasoning; they may be difficult for young children to perform accurately. The methodological demands of these tasks may partly explain the inconsistency of results across studies, highlighting the need to design a simpler, child-friendly task to more reliably assess comprehension of cognitive terms.

The term “forget” has two distinct meanings: “placing something and forgetting it” and “forgetting what one had remembered.” The latter refers to the opposite of “remembering,” and both reflect awareness of one's long-term memory. In contrast, “placing something and forgetting it” involves awareness of the effort to recall the moment of misplacement in order to retrieve the item. The term “know” refers to awareness of one's current state of knowledge. “Remember” judgments, which rely on conscious recollection, are cognitively more demanding than “know” judgments based on familiarity (e.g., Gardiner, 1988; Rajaram, 1993). Developmental studies have shown that while age-related differences are small for “know” judgments, “remember” judgments improve with age during elementary school (Piolino et al., 2007). These findings suggest that the term “know” may be understood earlier than “remember.” However, as the developmental course of cognitive terms in general remains unclear, the processes by which children conceptually distinguish between “know” and “remember” have yet to be fully elucidated. In the present study, the meaning of “forget” was restricted to “placing something and forgetting it,” and children's understanding of these terms was examined as they relate to three aspects of memory awareness. Importantly, the focus of the study is not on children's knowledge of the literal (lexical) meanings of these words, but rather on whether they understand the conceptual representation and application of these three aspects of memory states.

This study adopted new tasks to determine whether children understand the words “know” and “remember.” While Lockl and Schneider (2006, 2007) examined children's understanding of “forgetting” by asking them to consider what one should do after leaving a jacket behind at kindergarten, their task relied solely on verbal instructions and was difficult even for 6-year-olds. In contrast, our modified “forgetting” task supplemented the instructions with sequential illustrations and structured yes/no questions about concrete locations, making the task more accessible and producing clearer distinctions in children's understanding of “forgetting.” Similarly, the “remember” task was designed as a simple matching exercise: children judged whether a character's stated recollection (e.g., a pictured animal) was among the items they had just seen. This format minimized demands on memory and language ability, thereby providing a more direct assessment of children's grasp of “remember.” Based on prior findings (Uehara, 2014), we hypothesized that children would come to understand these concepts in the order of “know,” “forget,” and “remember,” typically between the ages of 4 and 6.

## 2 Methods

### 2.1 Participants

The participants were 38 4-year-olds [16 boys and 22 girls; mean age ± standard deviation (SD) = 4.33 ± 0.29 years; range: 3.82–4.87 years], 38 5-year-olds (20 boys and 18 girls; mean age ± SD = 5.42 ± 0.28 years; range: 4.88–5.84 years), and 38 6-year-olds (19 boys and 19 girls; mean age ± SD = 6.43 ± 0.30 years; range: 5.90–6.85 years) from a nursery school in a large city near Tokyo, Japan. All participants were monolingual native speakers of Japanese and, according to parental report, were typically developing children without confirmed developmental diagnoses.

### 2.2 Materials and procedures

Each participant was invited to a table in the corner of the classroom to complete three tasks: the “knowing,” “forgetting,” and “remembering” tasks. The total time required for all three tasks was approximately 10 min. For each task, participant responded to four yes/no questions. Because the “forgetting” and “remembering” tasks required additional explanation before the questions, the instructions were provided twice. If a child did not respond on the first attempt, the question was repeated once; all participants provided a response on either the first or second attempt for every item. A participant was classified as an “understander” of a given task only if they answered all four questions correctly. The order of the three tasks and of the four questions within each task was varied across participants in a quasi-random fashion. The figures used during the tasks were created using Microsoft Office 2007 and 2010 clip art, in accordance with Microsoft's End User License Agreement.

#### 2.2.1 “Knowing” task

The participant was shown an illustration depicting the faces of four child characters—Taro (boy), Jiro (boy), Kazuko (girl), and Hanako (girl)—on the left side, with each character's response displayed to the right of their face. The experimenter then pointed to each character in turn and asked the participant four questions. A sample outline of the illustration presented to the child is provided in Supplementary material S1. For example, the experimenter said: “*When I asked Taro, ‘What animals do you know?' Taro replied, ‘The animals I know are koalas, zebras, and pandas.' Is Taro's response correct?”* The participant was asked to judge whether each character's response was correct.

Each character's response contained two or three items. Two characters included one inappropriate item (e.g., *ramen* or *curry rice*), whereas the other two characters listed only animal names. The animal names used in this task were koala, panda, monkey, rabbit, bear, and zebra, all of which had been confirmed in advance to be familiar to the children. The order of the characters who responded incorrectly, as well as the content of each character's response, was pseudo-randomized across participants.

#### 2.2.2 “Forgetting” task

The participant was shown an illustration depicting Hanako with a backpack and a bag on the left, the nursery school in the center, and Hanako leaving the school carrying only her backpack and walking toward a car on the right. A sample outline of the illustration presented to the child is provided in Supplementary material S2.

The experimenter then explained: “*Hanako went to the nursery school, placed her bag in the X room (the participant's own classroom), and played there without visiting any other locations. After playing, Hanako left the school in her guardian's car. When she arrived home, she realized that she had left her bag behind. Where should Hanako look for it?”* Immediately afterward, the participant was asked four yes/no questions: (1) *Is it correct for Hanako to look in a park?* (Incorrect), (2) *Is it correct for Hanako to look in the X room at the nursery school?* (Correct), (3) *Is it correct for Hanako to look in the babies' room at the nursery school?* (Incorrect), and (4) *Is it correct for Hanako to look in her home?* (Incorrect).

#### 2.2.3 “Remembering” task

**Figure 1:**
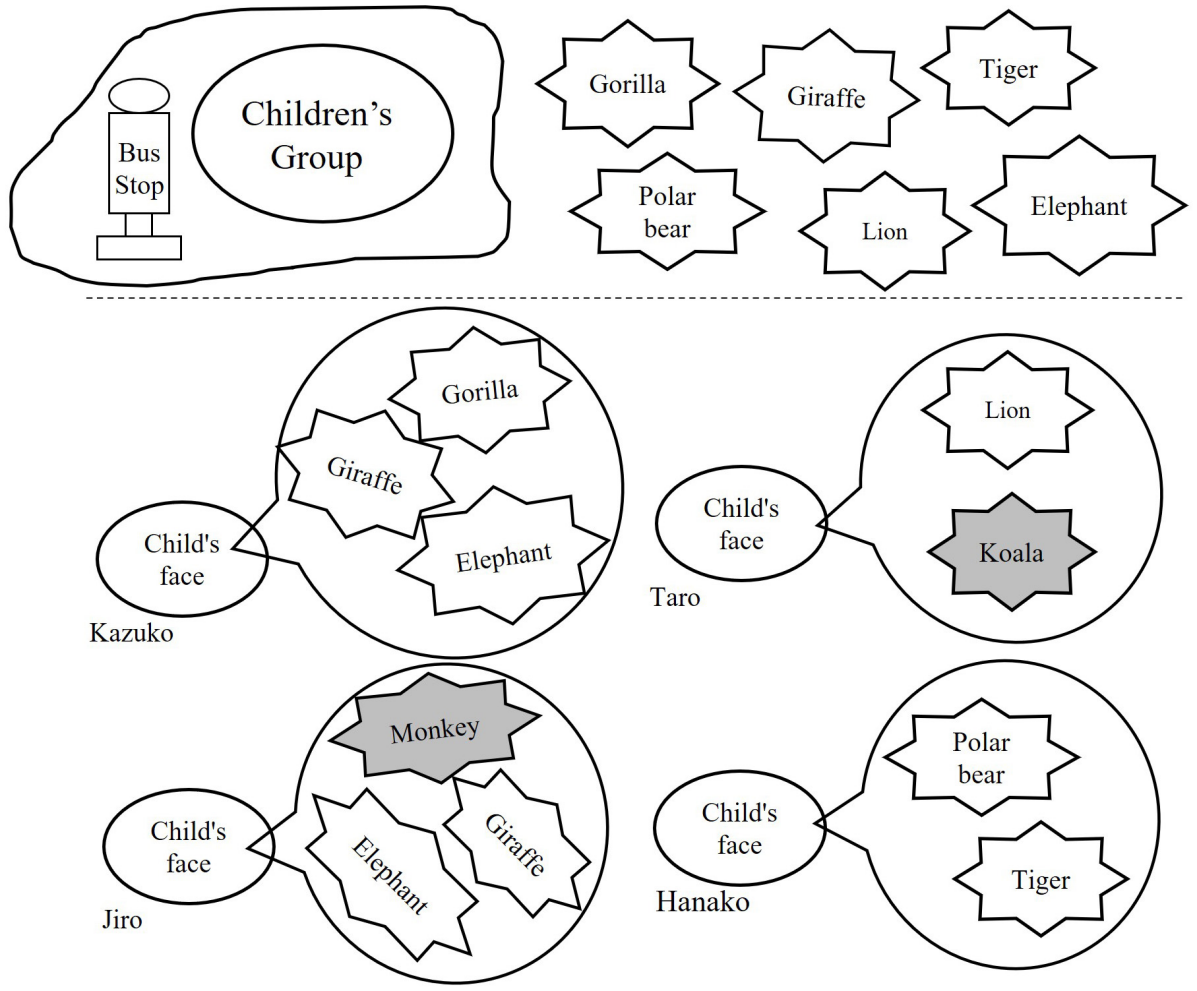
An outline of the illustration presented to the participant during the “remembering” task. In the lower part, each character and the animals in answer to the “remembering” question are illustrated. In this example, whether the participant could judge whether Kazuko and Hanako answered correctly and Taro and Jiro answered incorrectly was checked. 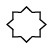: correct, 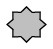: wrong.

The experimenter explained to the participant: “*All of the children went to the zoo by bus”* (while pointing to the four characters at the bottom of Figure 1, then to the group of children at the bus stop in the upper left). “*At the zoo, they saw six animals: a gorilla, a polar bear, a giraffe, a lion, a tiger, and an elephant”* (while pointing to each animal illustrated in the upper right of Figure 1).

Next, the experimenter described each character's recollection and asked whether the answer was correct. For example: “*When they returned home from the zoo, I asked Kazuko, ‘Kazuko, do you remember which animals you saw at the zoo? If you remember, please tell me.' Kazuko answered, ‘I remember seeing a giraffe, a gorilla, and an elephant.' Is Kazuko's answer correct?”* (while pointing to the content of Kazuko's response illustrated next to her face in Figure 1).

Each character's response listed two or three animals. Two characters included one inappropriate animal (either “koala” or “monkey”), while the other two named only animals that were actually shown in the zoo illustration (upper right of Figure 1).

The upper part of Figure 1 (the zoo scene) was identical across participants, whereas the lower part was varied such that the placement of characters' faces, the assignment of incorrect responses, and the specific animals included in each response were pseudo-randomized across participants.

This study was conducted under the supervision of the school. Written informed consent was obtained in advance from the parents of all participants, and the children took part voluntarily. All procedures in this study were reviewed and approved by the Humanities and Social Sciences Research Ethics Committee of Ochanomizu University (ethics approval number: 2017-103). The study was carried out in accordance with the principles of the Declaration of Helsinki.

### 2.3 Statistical analyses and use of large language models

Statistical analyses were performed using IBM SPSS Statistics, version 29 (IBM Corp., Armonk, NY, USA). In the original manuscript, the language was reviewed by two professional editors who were native English speakers. For the revised manuscript, all English paraphrasing and grammar refinement were conducted with the assistance of ChatGPT (a large language model developed by OpenAI) to ensure clarity and consistency throughout the text.

## 3 Results

As no significant gender differences were found in the proportion of understanders for the three tasks within each age group, data from boys and girls were analyzed together.

**Figure 2:**
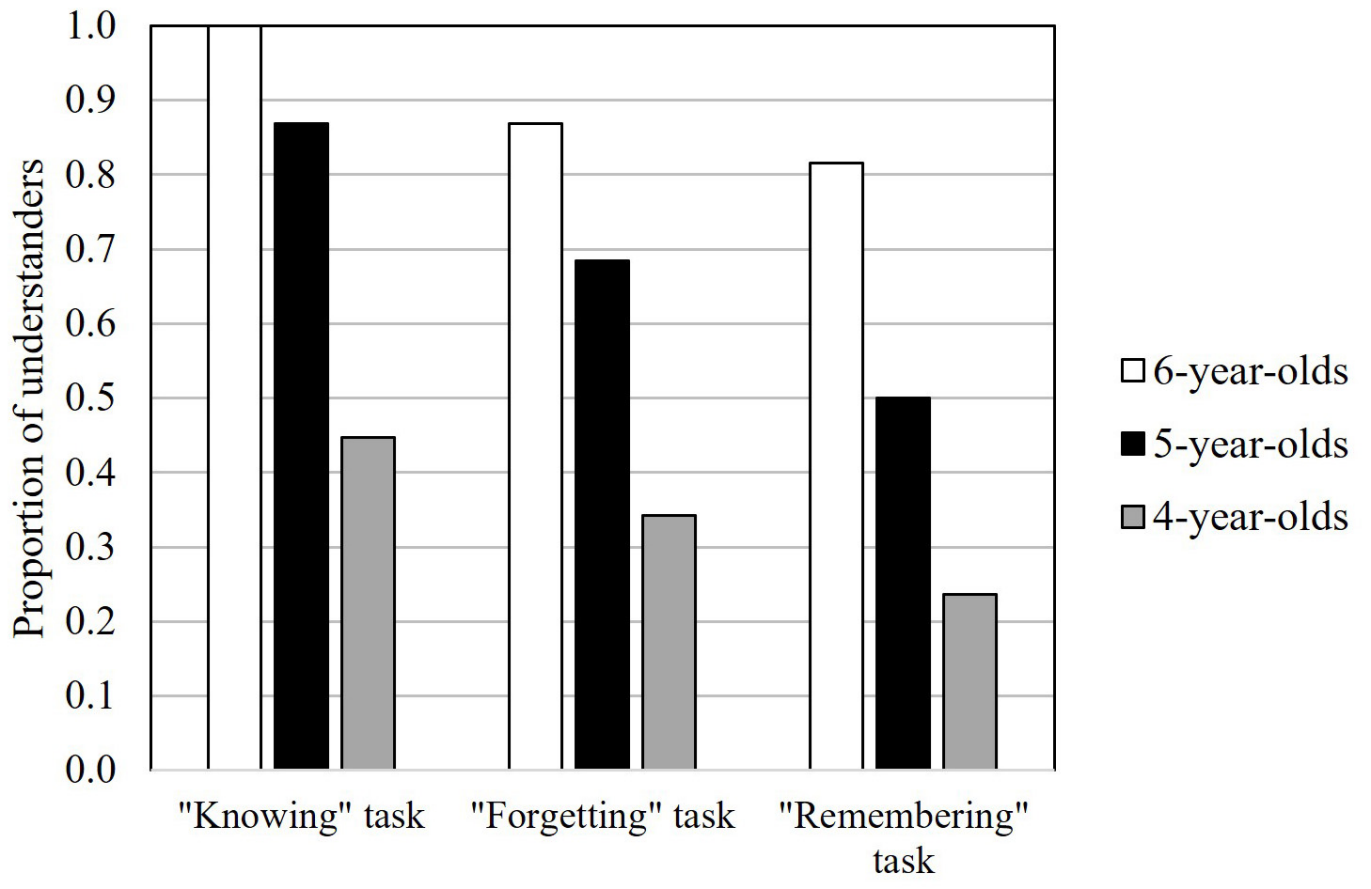
The proportion of understanders in each task with age group as a parameter.

Figure 2 presents the proportion of understanders for each task by age. Task performance was compared across the three age groups. In the “knowing” task, all 6-year-olds and 87% of 5-year-olds demonstrated comprehension, whereas only 45% of 4-year-olds did so, indicating significant age differences [χ^2^[2] = 36.00, *p* < 0.001, *V* = 0.562, power = 1.00; residual analyses identified four significant categories (*p* < 0.01): 4-year-olds' “understander” and “non-understander,” and 6-year-olds' “understander” and “non-understander”]. In the “forgetting” task, 87% of 6-year-olds and 68% of 5-year-olds demonstrated comprehension, compared with only 34% of 4-year-olds, again revealing significant age differences [χ^2^[2] = 23.30, *p* < 0.001, *V* =0.452, power =0.99; residual analyses indicating four categories significant (*p* < 0.01): 4-year-olds' “understander” and “non-understander,” and 6-year-olds' “understander” and “non-understander”]. In the “remembering” task, 82% of 6-year-olds and 50% of 5-year-olds demonstrated comprehension, whereas only 24% of 4-year-olds did so [χ^2^[2] = 25.58, *p* < 0.001, *V* = 0.474, power = 1.00; residual analyses again identified four significant categories (*p* < 0.01): 4-year-olds' “understander” and “non-understander,” and 6-year-olds' “understander” and “non-understander”].

The data were analyzed to explore whether children developmentally understand the mental states of knowing, forgetting, and remembering, in that order, based on the combined data from all age groups. A significant difference between the children who understood the “knowing” task and those who did not was found in the proportion of children who understood the “forgetting” task (χ^2^[1] = 50.92, *p* < 0.001, *V* = 0.668, power = 1.00), suggesting that children developmentally understand the “forgetting” state after the “knowing” state. A significant difference between the children who understood the “forgetting” task and those who did not was also found in the proportion of children who understood the “remembering” task (χ^2^[1] = 24.49, *p* < 0.001, *V* = 0.464, power = 1.00), suggesting that children developmentally understand the “remembering” state after the “forgetting” state.

## 4 Discussion

In sum, more than 80% of the 6-year-olds demonstrated an understanding of the three memory-related mental states, whereas the 4-year-olds did not. The 5-year-olds fell in between: about half did not understand the state of “remembering,” and approximately 30% did not understand the state of “forgetting.” Although the “knowing” task could, in theory, be solved through a simple category judgment of whether each item was an animal or not, this seems unlikely. Even 4-year-olds—who can easily distinguish animals from familiar foods such as curry rice or ramen—showed a relatively low correct response rate of about 45%. This suggests that children were attending to the character's statement containing the word *know* and were attempting to interpret its meaning, rather than responding based purely on categorical knowledge. These findings indicate that children's understanding develops sequentially, in the order of “knowing,” “forgetting,” and “remembering.”

This study also demonstrated the utility of newly developed tasks for assessing children's understanding of the mental states of “knowing” and “remembering.” The modified “forgetting” task, adapted from Lockl and Schneider's (2006, 2007) questions, was likewise shown to be effective. Unlike the original task, which relied solely on verbal instructions and primarily open-ended responses, our version incorporated sequential illustrations and structured yes/no questions targeting specific locations. These modifications made the task more accessible to preschoolers and provided clearer distinctions in their understanding of the concept of “forgetting.”

A clear developmental sequence in children's understanding of the three types of memory awareness was demonstrated. Few previous studies have identified developmental differences or clarified the order in which children acquire these three metamemory terms. The present findings are consistent with some prior work on “knowing.” Moore et al. (1989) showed that 5-year-olds distinguished the word “know” from “think” and “guess.” Similarly, Miscione, et al. (1978) reported that children younger than 4 years could not differentiate between “know” and “guess,” whereas most children older than 5 years and 5 months could.

However, some previous findings diverge from the present results. Although 68% of the 5-year-olds in this study demonstrated an understanding of “forgetting,” Lockl and Schneider (2006, 2007) found that the proportion of correct answers to forced-choice “forgetting” questions was very low for both 5½- and 6-year-olds. As noted by Lockl and Schneider (2007), the “forgetting” question in their task may have been particularly difficult, given that about two-thirds of the 5½-year-olds answered other memory-related questions correctly. Johnson and Wellman (1980) examined children's responses to “know,” “remember,” and “guess” questions and found that 4-year-olds showed little differentiation among these verbs, 5-year-olds began to distinguish them, first graders showed clearer distinctions, and full differentiation was not observed until third grade. In contrast, the present study demonstrated clear developmental progressions for “knowing,” “forgetting,” and “remembering” among 4-, 5-, and 6-year-olds. The three cognitive verbs may reflect different levels of metacognitive awareness: “knowing” relates to awareness of one's current knowledge state or familiarity-based judgments (e.g., Gardiner, 1988; Rajaram, 1993); “forgetting” involves awareness of the effort to recall a moment of misplacement or retrieval failure; and “remembering” relies on conscious recollection of past experiences, a cognitively demanding process (e.g., Gardiner, 1988; Rajaram, 1993). Given that autobiographical memory—an awareness of one's personal past—emerges around age 4 (Nelson and Fivush, 2004; Uehara, 2015), the observed developmental sequence (“knowing” → “forgetting” → “remembering”) appears theoretically coherent and consistent with prior research on the development of self-referential memory processes.

This study has several limitations that should be acknowledged. First, we did not administer a verbal working memory task, nor did we include multiple vignettes or trials that could have reduced measurement error and more reliably assessed the targeted conceptual understanding. Second, although the tasks tapped children's linguistic understanding of cognitive concepts, we did not administer standardized assessments of language ability. Such measures could have provided additional information about individual differences in language development; however, in the preschool context of this study, formal testing was not feasible because it could have created a sense of competition or comparison among children. Third, the sample was restricted to middle-class, monolingual, native Japanese-speaking children, which limits the generalizability of the findings. Future studies should address these issues by incorporating verbal working memory tasks, multiple vignettes and trials, standardized language measures, and more diverse socioeconomic and cultural groups, thereby strengthening both the reliability and validity of the tasks.

Although these limitations should be considered, further research building on the present findings has the potential to substantially advance this area of study. In particular, examining how children's comprehension of these linguistic tasks relates to performance on other cognitive measures across a wider age range could clarify the developmental processes underlying metamemory from preschool through the school years. Such work would not only enhance the validity of the tasks but also provide valuable insights for supporting children's cognitive development.

## Data Availability

The raw data supporting the conclusions of this article will be made available by the authors, without undue reservation.
