# Supplementary material for: Developmental sequence of young children's understanding of “knowing,” “forgetting,” and “remembering”
Source: Front Psychol. 2025 Nov 20;16:1626407. doi: 10.3389/fpsyg.2025.1626407 (PMC12676283; doi:10.3389/fpsyg.2025.1626407)
Supplement: Supplementary file 1 [file Data_Sheet_1.pdf]

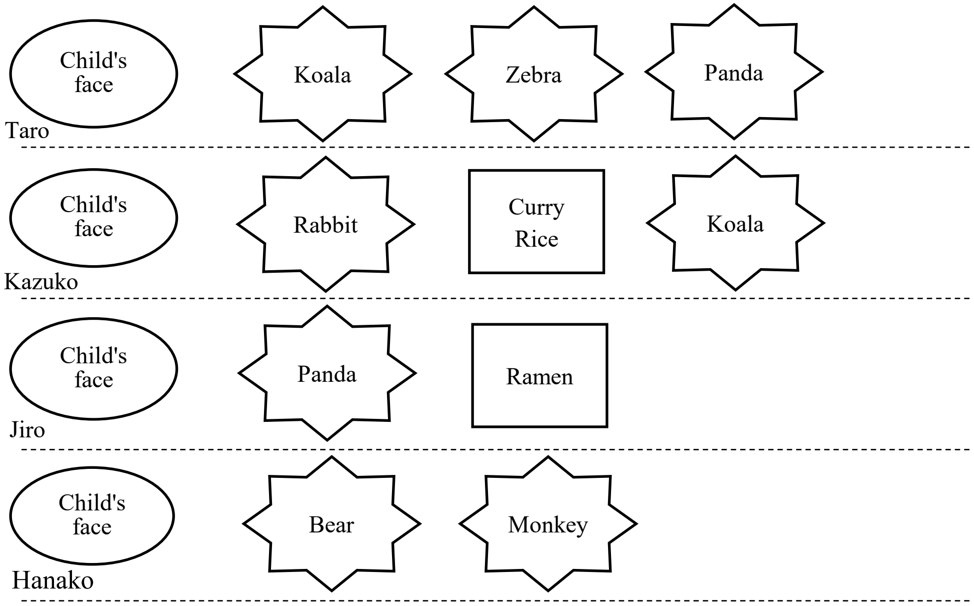

Figure S1. An outline of the illustration presented to the participant during the "knowing" task

Note: The faces of the four characters were lined up on the left, and next to them was an illustration of what the character had answered to the "knowing" question. In this example, the task question involved whether the participants could judge that Taro and Hanako had answered correctly and that Kazuko and Jiro had answered incorrectly. ✡: correct, □: wrong
