# Supplementary material for: Developmental sequence of young children's understanding of “knowing,” “forgetting,” and “remembering”
Source: Front Psychol. 2025 Nov 20;16:1626407. doi: 10.3389/fpsyg.2025.1626407 (PMC12676283; doi:10.3389/fpsyg.2025.1626407)
Supplement: Supplementary file 2 [file Data_Sheet_2.pdf]

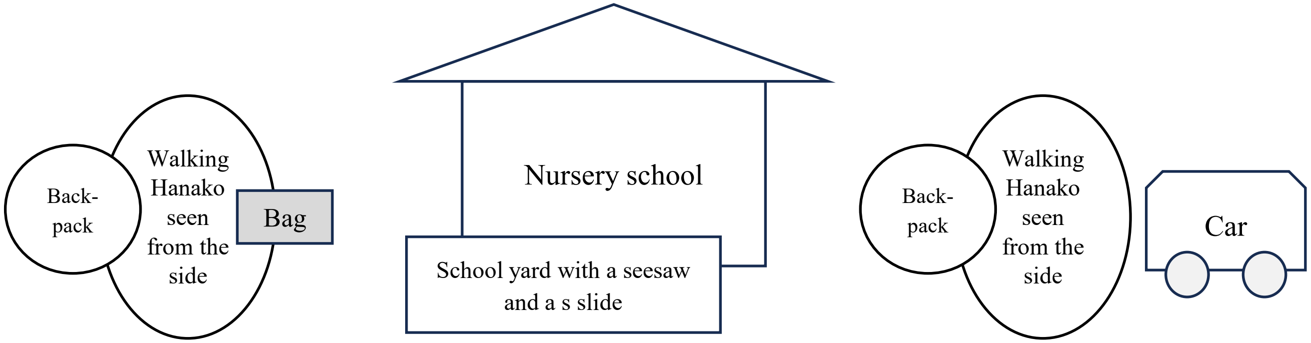


**Figure S2. An outline of the illustration presented to the participant during the "forgetting" task**

Note: A character named Hanako carrying a backpack together with a bag on the left side of the card, the nursery school in the center, and Hanako carrying only her backpack to a car that was taking her home on the right side.
